# Does *Becoming* Fit Mean *Feeling* (f)it? A Comparison of Physiological and Experiential Fitness Data From the iReAct Study

**DOI:** 10.3389/fspor.2021.729090

**Published:** 2021-09-01

**Authors:** Hannes Gropper, Felipe Mattioni Maturana, Andreas M. Nieß, Ansgar Thiel

**Affiliations:** ^1^Department of Social and Public Health Sciences, Institute of Sport Science, Eberhard Karls University Tübingen, Tübingen, Germany; ^2^Interfaculty Research Institute for Sport and Physical Activity, Eberhard Karls University Tübingen, Tübingen, Germany; ^3^Department of Sports Medicine, University Hospital Tübingen, Tübingen, Germany

**Keywords:** exercise, high-intensity interval training, moderate-intensity continuous training, physical fitness, physiology, subjective experience, interdisciplinary, mixed-methods

## Abstract

Regular exercise fosters fitness-enhancing benefits. We assume that exercise interventions become successful and sustainable if physiological benefits of exercise are also subjectively perceivable. The goal of this study was to examine how young inactive adults *physiologically respond* to an exercise intervention and how those responses are *subjectively experienced*. Furthermore, we aimed to assess whether the sequence of two distinct endurance-based exercise modes has an impact on physiological and subjectively experienced physical fitness. Thirty-one young inactive adults were assessed for this substudy of the larger iReAct study. Participants were randomly assigned to a high-intensity interval training (HIIT) or a moderate-intensity continuous training (MICT) group for 6 weeks and subsequently switched groups for a second training period. Physiological fitness data was collected at baseline, follow-up I, and follow-up II using a graded cardiopulmonary exercise test. Subjectively reconstructed (i.e., retrospective constructions) experiences relating to physical fitness were assessed at follow-up II using a biographical mapping method. A repeated-measures one-way ANOVA on each training group was performed to see whether physiological and subjectively experienced fitness differed across training periods. The rate of change between all variables was calculated for the first and the second training period in order to compare the agreement between physiological and subjective fitness improvements. Participants increased their fitness across the intervention period both physiologically and subjectively. However, the rate of change depended on the sequence of the two training modes. While VO_2max_ increased significantly in both training periods in the MICT-HIIT sequence, a significant increase in VO_2max_ in the HIIT-MICT sequence was only observed in the HIIT period. Participants similarly perceived those increases subjectively in their exercise-related physical fitness, although they experienced a significant decrease in the second period of the HIIT-MICT sequence. For subjectively perceived physical fitness relating to everyday activities, significant increases were only observed for the first period of the MICT-HIIT sequence. Young inactive adults can improve both their physiological and their subjectively perceived fitness through regular exercise. However, exercise modes and their sequence can make a substantial difference regarding measured and perceived physical fitness. Additionally, despite a favorable tendency toward HIIT over MICT, inter- and intra-individual variability, particularly in the subjective experiences of fitness, reiterates the necessity of individualized approaches to exercise.

## Introduction

Researchers have described exercise as a “polypill” acknowledging its positive impact on biopsychosocial health and the decisive role it has come to play in both prevention and therapy (Fiuza-Luces et al., 2013; Rebelo-Marques et al., 2018). While dose-response-effects of exercise on health and fitness are well-established on a physiological level (Maturana et al., 2020), it is less clear whether and to which extent individuals who exercise also *subjectively experience* those changes in their physical fitness over time.

While the health-enhancing benefits of regular physical activity (PA) and exercise are vastly documented (Rhodes et al., 2017; Warburton and Bredin, 2017), even the largest public health initiatives have so far been insufficiently successful in motivating people to meet internationally recommended minimum levels of regular activity, as indicated by studies on the prevalence of physical (in)activity (Hallal et al., 2012; Guthold et al., 2018). One reason might be a lack of knowledge about how physiological training effects of exercise correlate with the subjective experience of exercise. We assume that a successful and sustainable structured exercise training requires that the physiological benefits of exercise must also be subjectively perceivable. While several studies that assessed the relationship between actual (i.e., “objectively” measured) and perceived (i.e., self-reported) physical fitness report significant correlations particularly for cardiorespiratory fitness measures and self-perceptions of endurance and fitness (Germain and Hausenblas, 2006; Monroe et al., 2010), there are no studies that examine how *physiological adaptations* to an exercise intervention relate to *subjective perceptions* of these adaptations over time.

Given the lack of studies on the relationship between objectively measurable physiological adaptations and the subjective experience of exercise outcomes across an intervention period, interdisciplinary biopsychosocial approaches, which integrate physiological and experiential factors over time, are needed (John et al., 2020). In this regard, it is interesting to examine the processual question of whether improvements in physiological fitness make individuals actually feel fitter. Answering this question is highly relevant, because we assume that even a high degree of physiological responsiveness to training has a larger effect on the individual if this response is also subjectively palpable. Eventually, if individuals do not *feel* the progress of becoming fitter, this lack of a subjectively beneficial response might jeopardize a long-term exercise engagement.

In the search for optimal strategies to counteract a prevailing trend toward more sedentary and inactive lifestyles, a controversial discussion on the use of different exercise modes has evolved. One of the focus points in this debate is the role of high-intensity interval training (HIIT) as a viable option for public health strategies (Biddle and Batterham, 2015) as opposed, for example, to moderate-continuous training (MICT). In this regard, the physiological benefits and the time efficacy of HIIT are well-documented (Gibala et al., 2014; Biddle and Batterham, 2015; Maturana et al., 2020). Moreover, research on psychological and affective responses to HIIT have indicated that interval training might be as (Stork et al., 2017, 2018) or even more enjoyable or preferable than MICT (Jung et al., 2014; Stork et al., 2017). We can therefore assume that HIIT might trigger different physiological and psychological effects than MICT.

The goal of our paper is to assess how individuals *physiologically respond* to HIIT in comparison to MICT and how those responses are *subjectively experienced and perceived* over time. In addition, we think that prescribing different sequences of training modes (i.e., HIIT first and MICT second or vice versa), might lead to different subjective experiences of the same training mode, because participants can comparatively evaluate, which training mode they perceive to be more beneficial.

Against this background, we aim to answer the following questions:

1. How do physiological fitness and subjective experiences of fitness develop over the time of an exercise intervention?
2. Does the sequence of two distinct endurance-based exercise programs (i.e., HIIT-MICT vs. MICT-HIIT) have an impact on how physiological fitness and subjective experiences of feeling fit develop?
3. How do physiological adaptations to exercise relate to subjective experiences of fitness?

## Materials and Methods

### Study Design and Setting

Our study is part of the *Individual Response to Physical Activity (iReAct)* project, which is a transdisciplinary research program that aims to assess individual responses to exercise in physically inactive young adults from an integrated biopsychosocial perspective (Thiel et al., 2020a). The project includes researchers from the fields of sport sociology, psychology, and medicine, as well as from biochemistry and psychosomatics and thus follows a multi-method approach. In this regard, one of the primary goals of the research program is to mesh objective and subjective data and therefore to provide a new perspective on the effectiveness of exercise programs and to contribute to the discussion about the necessity of individualized approaches.

After approval by the “Ethics Committee of the Medical Faculty University Tübingen” (reference number: 882/2017BO1), participants were recruited via several means including the university and the university clinics mailing lists, an experimental database, newspaper articles, and flyers. In order to participate, interested parties had to…

- …be physically inactive (i.e., currently not meeting the WHO's recommendations for moderate physical activity of at least 150 min/week and exercising for <60 min/week and not regularly exercising for several weeks during the last 6 months).
- …be non-smokers.
- …have a BMI between 18.5 and 30.0 kg/m^2^.
- …have a maximum oxygen uptake (VO_2max_) between 25 and 50 mL/min/kg.
- …have no current or former eating disorder.
- …have no severe internistic or neurological previous illness.
- …be not pregnant or being breastfeeding.
- …be German native-speakers.

**Figure 1:**
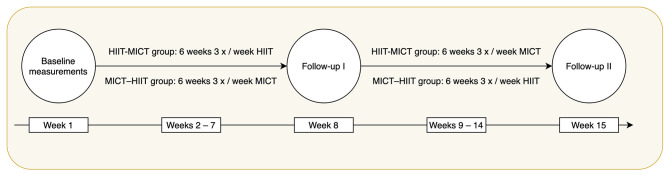
Timeline and protocol of the iReAct study.

Inclusion and exclusion criteria were chosen to ensure that inactive, yet only healthy, young adults were included. Young adults were selected as they find themselves in a period of their life were PA levels usually tend to drop due to various life events and transitions (Corder et al., 2020; Gropper et al., 2020; Winpenny et al., 2020) and therefore present a potentially “vulnerable” group for physical inactivity. Moreover, we opted for a healthy sample for two reasons: (1) PA and exercise are highly relevant in the prevention of non-communicable diseases. In order to have a preventive effect exercise should ideally be taken up while people are still healthy. (2) Participants should be able to sustain the study protocol. In addition, the inclusion and exclusion criteria accounted for the variety of biopsychosocial assessment tools (including various questionnaires and interviews that were in German) that were used [for more information and the detailed recruitment process, please see Thiel et al. (2020a)]. All participants were informed of the experimental protocol and all associated risks prior to giving written informed consent and being included in the study. The study was designed as a 15-week, two-period sequential-training intervention in order to compare adaptive responses to two training modes (HIIT vs. MICT) and their different sequential order (MICT-HIIT vs. HIIT-MICT). Biopsychosocial assessments took place on 3 days at baseline (week 1), after the first training period (week 8), and after the second training period (week 15). After the baseline assessment, participants were randomly assigned based on VO_2max_ and sex to either the HIIT or the MICT group in the first training period and switched groups for the second period. Each training period lasted 6 weeks with three training sessions per week. For a detailed overview of the study timeline, please see Figure 1.

A total of 58 participants were assessed for eligibility, 49 of whom were included in the randomization process. After baseline measurements, 44 participants engaged into the exercise training intervention (HIIT-MICT *n* = 22 and MICT-HIIT *n* = 22). One female, who exhibited a BMI below the specified range was also included due to her normal percent body fat of 23.5 (normal range: 18–28%) as measured at baseline. During the first training period, two participants (one from each group) dropped-out during the exercise training intervention due to illness and not being able to complete the minimum adherence (i.e., 15 out of 18 sessions in each training period). A total of 42 participants (21 in each group) completed the first follow-up. During the second training period, a total of 9 participants (4 from the HIIT-MICT group, and 5 from the MICT-HIIT group) dropped-out because they were unable to complete the minimum adherence due to the lockdown imposed by COVID-19. Two participants were excluded *post-hoc* due to language issues (one from each group). Therefore, a total of 31 participants completed the full protocol: 16 participants in the HIIT-MICT group, and 15 participants in the MICT-HIIT group.

### Exercise Training Intervention

HIIT and MICT were designed with the goal that both interventions would be matched by energy expenditure (Andreato, 2020). After careful consideration, we prescribed the following exercise training programs:

*HIIT:* the HIIT group performed 10 min of warm-up at the power output corresponding to 70% of their maximal heart rate (HR_max_), followed by four 4-min intervals at the power output corresponding to 90% of their HR_max_. Each high-intensity interval was interspersed with a 4-min active recovery at 30 W. After the last high-intensity interval a 5-min cool-down at 30 W was performed, totaling 43 min of exercise. The power output at each percentage of HR_max_ was derived from the heart rate-power output-relationship during the step incremental test performed at baseline (first training period) or the first follow-up (second training period). To account for the delay in the heart rate response to the increase in work rate in each step, the average of the last 30 s of each step was taken, and then plotted against power output, deriving the linear model used for the calculation. All of the exercise intensities associated with 90% of HR_max_ were greater than the intensity associated with the second lactate threshold (LTP2), confirming that such intensity was in the severe-intensity domain.

*MICT:* the MICT group performed 60 min of continuous cycling at the power output corresponding to 90% of the first lactate threshold (LTP1). LTP1 was analyzed as the first systematic increase in blood lactate concentration above baseline levels, and such exercise intensity was prescribed for participants to cycle within the moderate-intensity domain.

*Training monitoring:* all exercise training sessions were performed on a cycle ergometer (ec5000, custo med GmbH, Ottobrunn, Germany) and participants' heart rate and ECG were constantly monitored (3-channel ECG, custo med GmbH, Ottobrunn, Germany). After every training session, the exercise training data (i.e., second-by-second power output, cadence, and heart rate) was exported and stored for further processing. The heart rate data was cleaned using an anomaly detection algorithm to delete noisy data points. Each noisy data point was deleted, and the heart rate was then interpolated in a second-by-second basis. Once the training sessions of every week were completed (*n* = 3), the sessions were then ensembled-averaged and then averaged into 5-s bins. In this way, we retrieved one averaged dataset for each week. Thereafter, the weekly average response was compared with zones of smallest worthwhile difference around the prescribed heart rate [for further details, please see (Maturana et al., 2021)].

*Minimum adherence:* in order for participants to be included in the final analyses, a minimum of 15 out of the 18 prescribed exercise sessions in each training period had to be completed (minimum adherence = 83.3%).

### Data Sources

In order to answer our research questions, mixed-method approaches are needed, as we aim to compare repeated measures of physiological and subjectively experienced fitness over time. The combination of physiological and experiential data allows painting a contextualized picture of an understudied phenomenon. In this regard, our analyses can reveal general biological developmental tendencies and relate them to subjective perceptions of this development.

#### Physiological Data

Physical fitness was tested at baseline, follow-up I, and follow-up II using a graded cardiopulmonary exercise test, where gas exchange and blood lactate concentration measures were taken. Fitness was defined via cardiorespiratory fitness (CRF) through VO_2max_. After arrival in the laboratory, participants were directed to the room where anthropometrical measures were taken, which included height and weight (measured to the nearest 0.01 cm and 0.1 kg, respectively). Afterwards, participants undertook a step-incremental test to volitional exhaustion on a cycle ergometer (Ergoselect 200, Ergoline GmbH, Bitz, Germany) for determination of VO_2max_, peak power output, and lactate thresholds (LTP1 and LTP2). Before starting the test, capillary blood lactate concentration ([La^−^]) was measured. The test began with a 2-min resting period on the bike, followed by 25-W step increments every 3 min, starting at 50 W for males and at 25 W for females, until task failure. [La^−^] was analyzed (Biosen S-Line, EKF, Cardiff, UK) by collecting capillary blood samples (20 μL) from the right earlobe during the last 20 s of each stage and immediately after volitional exhaustion. Heart rate and ECG were constantly monitored throughout the test (12-channel PC ECG, custo med GmbH, Ottobrunn, Germany). Breath-by-breath pulmonary gas exchange and ventilation were measured using a metabolic cart (MetaLyzer, CORTEX Biophysics, Leipzig, Germany). Calibration was performed before each test following the manufacturer's instructions.

#### Experiential Data

At follow-up II, we assessed subjectively reconstructed (i.e., retrospective constructions) experiences of the intervention. This approach is rooted in constructivist biography-theoretical concepts (for an overview, please see Schubring et al., 2019). The basic assumption of these concepts is that an individual's judgement toward a given context or situation is informed by previous experiences and their reconstruction. In order to assess contextualized subjective experiences (e.g., in a specific exercise setting), we used a biographical mapping method. This method combines a narrative format with a drawing activity eliciting both qualitative and quantitative data on the subjectively reconstructed experiences and trajectories of 14 state and developmental variables that relate to physical activity, exercise, and health (Schubring et al., 2019; Thiel et al., 2020b). For a detailed overview of the assessed variables, please see Supplementary Table 1.

The core feature of the biographical mapping is a two-dimensional coordinate system with a time axis (x-axis) that represents the time course of the intervention period and a perceived intensity scale (y-axis) from zero to ten. In a first step, participants were asked about any relevant life events that occurred over the 15 weeks of the training intervention. The interviewer wrote down those events on the time axis and encouraged participants to talk about everything they considered to be important with regard to the last weeks. To give the participants some temporal orientation all training sessions were already pre-registered (e.g., to see when trainings were missed or when the follow-up I diagnostic took place). Secondly, participants drew developmental curves for various variables including one for perceived exercise-related physical fitness (i.e., training status or athletic fitness) and one for perceived physical fitness in everyday life (i.e., general fitness for everyday activities). In this context, the y-scale of the coordinate system represented an intensity scale, where participants could indicate whether certain variables were experienced as very high (i.e., 10) or very low (i.e., zero) at different times over the last weeks. In a similar vein, participants could indicate how these experiences developed over time (e.g., stable trajectories, abrupt or gradual changes, linearity, etc.). The curves represent a quantified account of retrospective and subjective experiences and are complemented by verbal accounts. For the present study, however, we focused our analysis on the curve trajectories. For an exemplary biographical mapping, please see Supplementary Figure 1.

To collect data we used the bioMAP-software (Thiel et al., 2020c,d). During the mapping, the participants sat in front of a large touchscreen, on which they could draw the curve trajectories with a stylus. For each day of the intervention period, the software obtained one data point. The researcher sat right next to the participants with a separate monitor to follow up on what they were drawing. In addition, the participants gave their consent to audiotape the interviews. HG, who, afterwards, took field notes and filled in a protocol sheet, conducted all interviews.

### Data Analysis

#### Analyzing Physiological Data

Breath-by-breath oxygen uptake (VO_2_) data were edited as follows: breath data points that were outside the 95% of confidence interval from the local mean were considered outliers and then removed. Thereafter, the data was interpolated on a second-by-second basis and averaged into 30-s bins for VO_2max_ analysis (Mattioni Maturana et al., 2018; Martin-Rincon et al., 2019).

##### Maximal Values

VO_2max_ was considered as the highest 30-s VO_2_ average. VO_2max_ attainment was confirmed if at least two of the following three criteria were met, as per the American College of Sports Medicine guidelines (American College of Sports Medicine et al., 2018): (i) maximal heart rate within 10 beats per minute (bpm) of the maximal predicted value (220–age); (ii) a respiratory exchange ratio (RER) higher than 1.10; or (iii) a maximal [La^−^] of 8 mmol·L^−1^. Peak power output (PO_peak_) was considered as the power output achieved at the moment of exhaustion and HR_max_ was considered as the maximal value achieved during the test.

##### LTP1 and LTP2

Lactate thresholds were analyzed using a segmented regression model at which two breakpoints were estimated from the [La^−^]-power output relationship. LTP1 was determined as the first rise in [La^−^] above baseline levels (first breakpoint), which is accompanied by the first systemic increase in the VE-VO_2_ relationship (i.e., first ventilatory threshold). LTP2 was determined as the second abrupt increase in [La^−^] (second breakpoint), which is accompanied by the second sharp increase in the VE-VO_2_ relationship (i.e., second ventilatory threshold) (Binder et al., 2008; Hofmann and Tschakert, 2017). All these measures were analyzed as a function of power output, and then their corresponding VO_2_ values were analyzed from the VO_2_-power output relationship.

#### Statistical Analysis of Physiological and Experiential Fitness Trajectories

Normality (Shapiro-Wilk's test), homoscedasticy (Levene's test), and multicollinearity were checked when applicable. To calculate whether the continuous variables (i.e., VO_2max_, perceived exercise-related physical fitness, and perceived physical fitness in everyday life) differed across training phases (i.e., baseline, follow-up I, and follow-up II), we performed a repeated-measures (within-subjects) one-way analysis of variance (ANOVA) on each training group (i.e., HIIT-MICT and MICT-HIIT). Effect sizes were calculated as the partial omega-squared, and multiple comparisons were adjusted by the Bonferroni correction. The alpha level was set at 0.05.

In order to compare and relate the changes in VO_2max_ to the changes in the subjective fitness levels (indicated by the biographical mapping curves for perceived exercise-related physical fitness and perceived physical fitness in everyday life), we calculated the slopes of the changes in each training period (i.e., from baseline to follow-up I and from follow-up I to follow-up II) and in each group (i.e., HIIT-MICT and MICT-HIIT). We then performed Pearson correlations, adjusted for multiple comparisons in each training phase. For the VO_2max_ slopes, a simple linear regression between two data points was performed (i.e., from baseline to follow-up I and from follow-up I to follow-up II). For the subjective fitness levels, since continuous data were obtained (i.e., one data point for each day during the training period), we performed a linear regression from the first until the last training day. Additionally, we performed a linear regression to calculate the rate of change in the subjectively perceived fitness levels in relation to the rate of change in VO_2max_.

## Results

**Figure 2:**
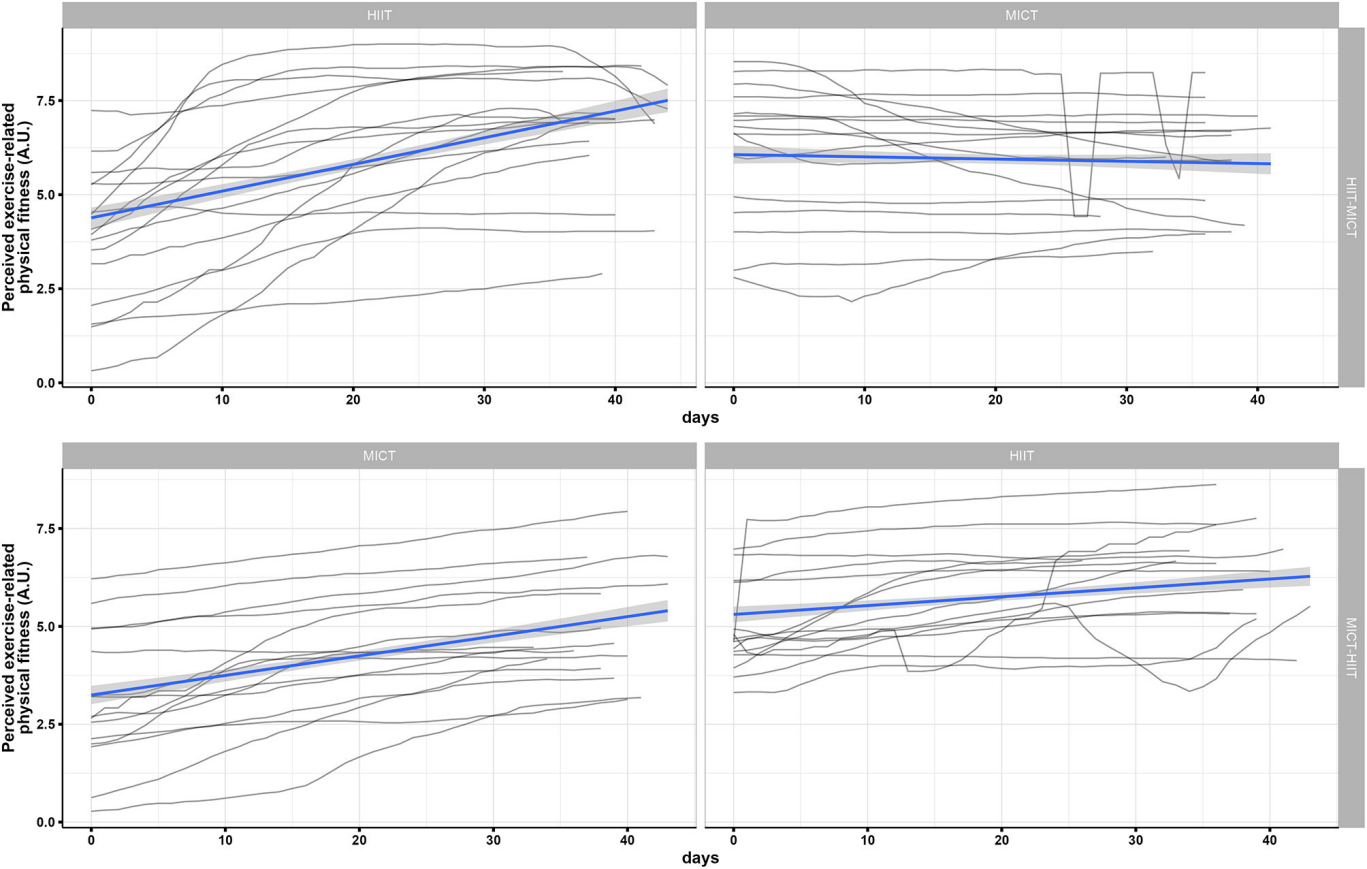
Individual mapping trajectories and mean trajectory of perceived exercise-related physical fitness over the course of each training period and group.

**Figure 3:**
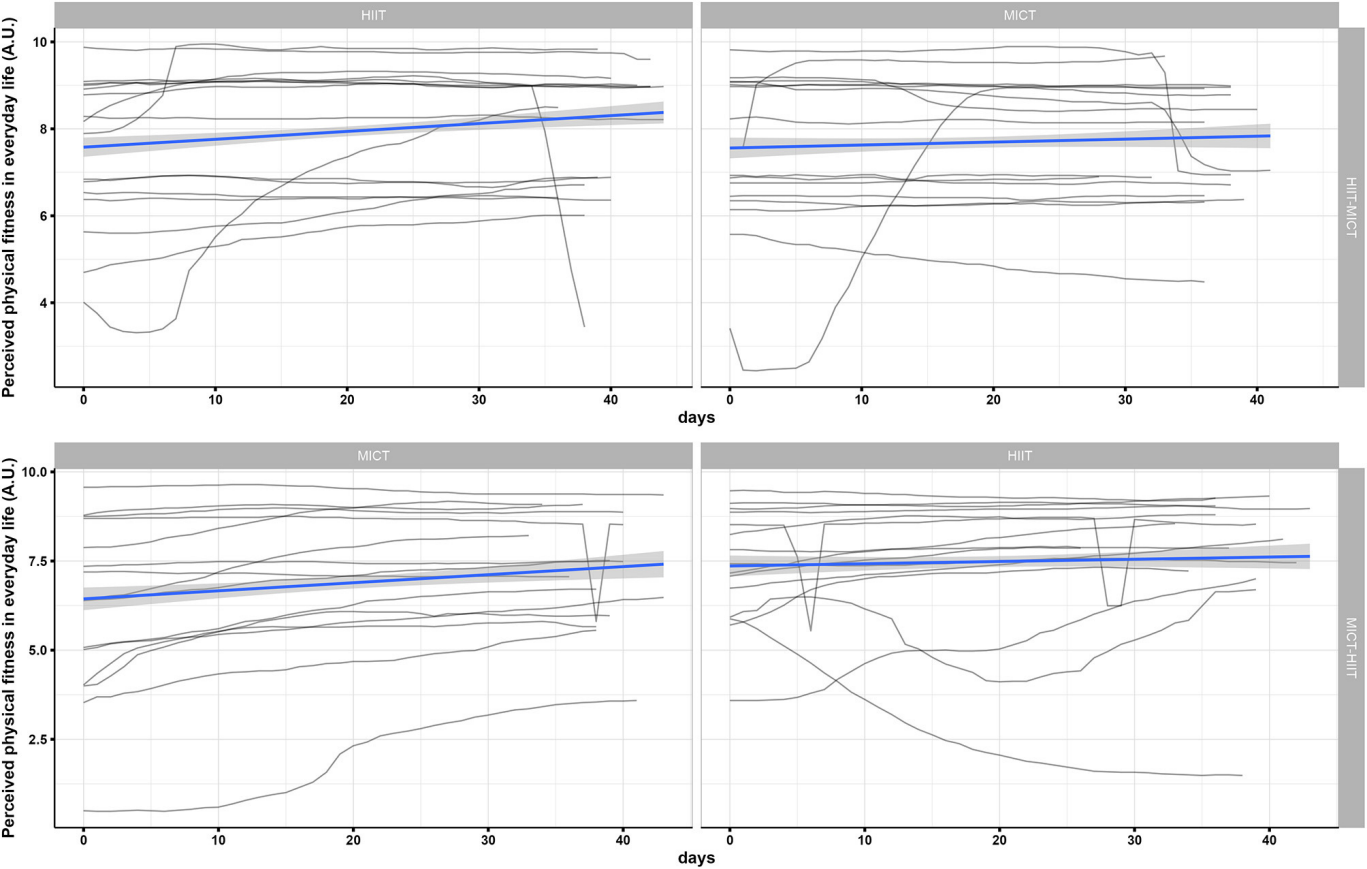
Individual mapping trajectories and mean trajectory of perceived physical fitness in everyday life over the course of each training period and group.

Data was collected from 31 participants (74% female) aged between 20 and 40 years (M = 27, SD = 6). For an overview of participants' characteristics at baseline in each group, please see Table 1. Figures 2, 3 display the inter- and intra-individual variability in the subjectively perceived fitness measures over the course of each training period and group over the 6 weeks of training.

**Table 1:** Participants' characteristics at baseline in each group.

	**HIIT-MICT**	**MICT-HIIT**	***p*-value**
Age (yr)	26 ± 5	29 ± 6	*p =* 0.127
Height (cm)	171.0 ± 9.2	170.9 ± 8.6	*p =* 0.991
Weight (kg)	68.1 ± 12.2	71.0 ± 8.9	*p =* 0.382
Absolute VO_2max_ (L/min)	2.14 ± 0.48	2.15 ± 0.36	*p =* 0.955
Relative VO_2max_ (mL/kg/min)	31.5 ± 4.4	30.4 ± 4.3	*p =* 0.417
PO_peak_ (W)	162 ± 29	159 ± 21	*p =* 0.685
HR_max_ (bpm)	190 ± 10	191 ± 12	*p =* 0.764
SBP rest (mmHg)	117 ± 9	115 ± 9	*p =* 0.645
DBP rest (mmHg)	78 ± 6	77 ± 6	*p =* 0.586
SBP max (mmHg)	154 ± 14	150 ± 14	*p =* 0.407
DBP max (mmHg)	66 ± 9	65 ± 7	*p =* 0.493

*VO_2max_, maximal oxygen uptake; PO_peak_, peak power output; HR_max_, maximal heart rate; SBP, systolic blood pressure; DBP, diastolic blood pressure*.

**Figure 4:**
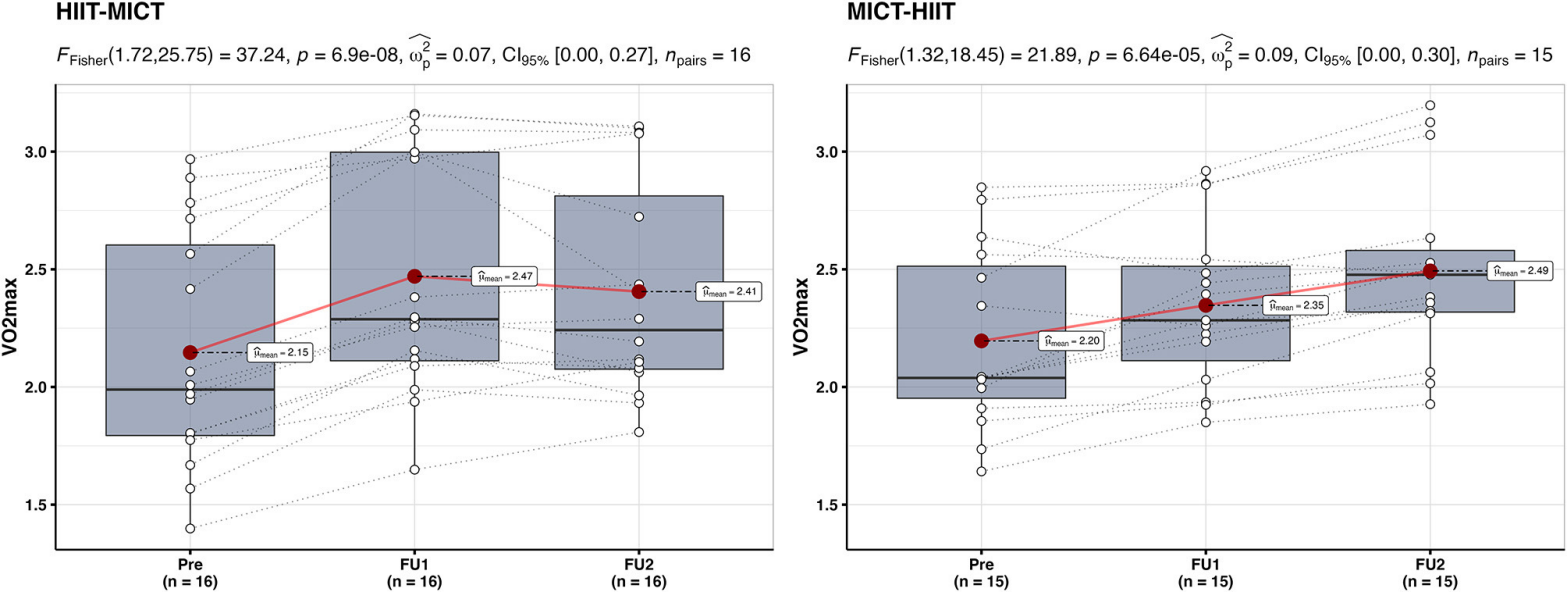
Within-group between baseline (Pre), follow-up I (FU1), and follow-up II (FU2) in the HIIT-MICT and MICT-HIIT groups for VO_2max_. Boxplots along with the individual data points are shown. The dotted lines connect the same participant, displaying the individual changes. The red dots display the average of each time point.

The repeated measures ANOVA for VO_2max_ (Figure 4) indicated a significant effect for both the HIIT-MICT {*F*_(1.72, 25.75)_ = 37.24, *p* < 0.001, ω^2^ = 0.07, 95% CI = [0.00; 0.27]} and MICT-HIIT groups {*F*_(1.32, 18.45)_ = 21.89, *p* < 0.001, ω^2^ = 0.09, 95% CI = [0.00; 0.30]}. Pairwise *post-hoc* analyses showed a significant difference from baseline to follow-up I in the HIIT-MICT group after HIIT (+ 16.0 ± 7.3%, *p* < 0.001), but no significant differences were found from follow-up I to follow-up II (after MICT, −0.02 ± 7.3%). In the MICT-HIIT group, however, significant differences were found in both training phases—from baseline to follow-up I after MICT (+7.7 ± 8.8%, *p* = 0.022) and from follow-up I to follow-up II after HIIT (+ 6.2 ± 4.0%, *p* < 0.001).

**Figure 5:**
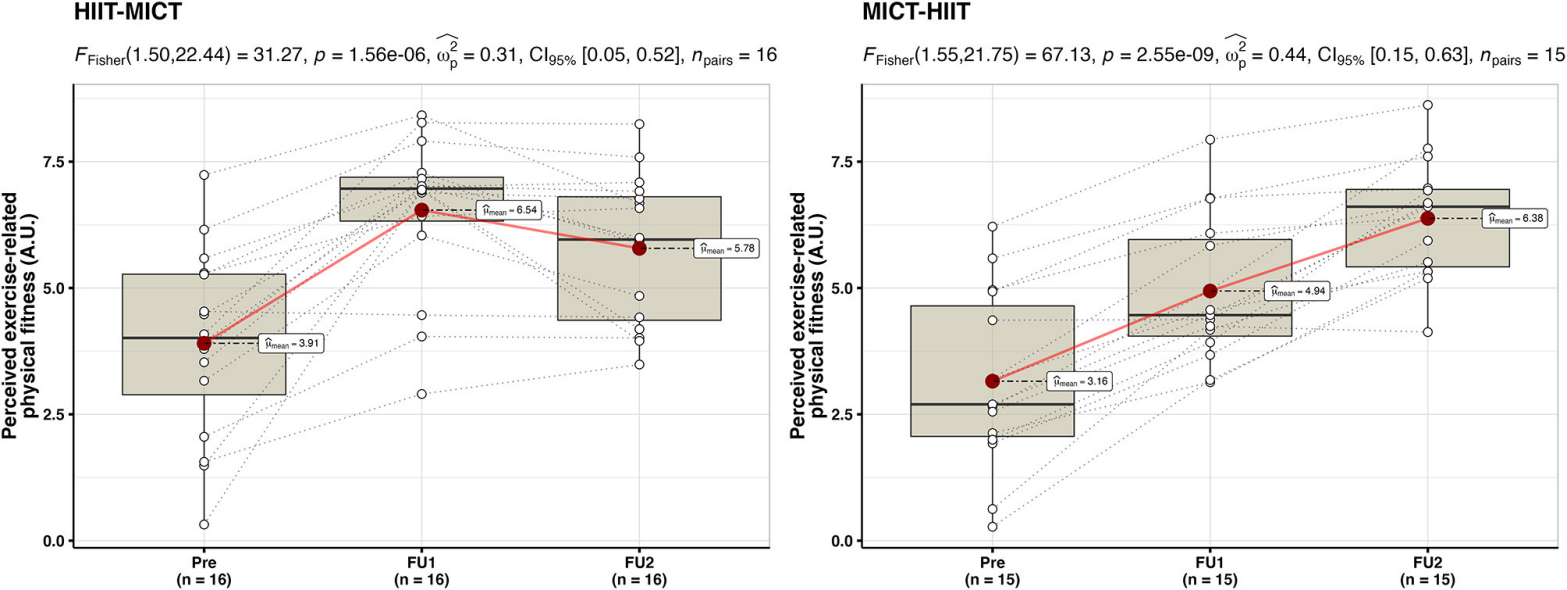
Within-group between baseline (Pre), follow-up I (FU1), and follow-up II (FU2) in the HIIT-MICT and MICT-HIIT groups for perceived exercise-related physical fitness. Boxplots along with the individual data points are shown. The dotted lines connect the same participant, displaying the individual changes. The red dots display the average of each time point.

The repeated measures ANOVA for perceived exercise-related physical fitness (Figure 5) showed similar results as VO_2max_. The HIIT-MICT presented a significant main effect {*F*_(1.50, 22.44)_ = 31.27, *p* < 0.001, ω^2^ = 0.31, 95% CI = [0.05; 0.52]}, with a significant increase from baseline to follow-up I after HIIT (+ 204 ± 505%, *p* < 0.001). Interestingly, a significant decrease from follow-up I to follow-up II after MICT was identified (−10 ± 16%, *p* = 0.035), while VO_2max_ did not change. The MICT-HIIT group also presented a significant main effect {*F*_(1.55, 21.75)_ = 31.27, *p* < 0.001, ω^2^ = 0.44, 95% CI = [0.15; 0.63]}, with a significant increase in both training phases (MICT: + 154 ± 283%, *p* < 0.001; HIIT: + 35 ± 28%, *p* < 0.001).

**Figure 6:**
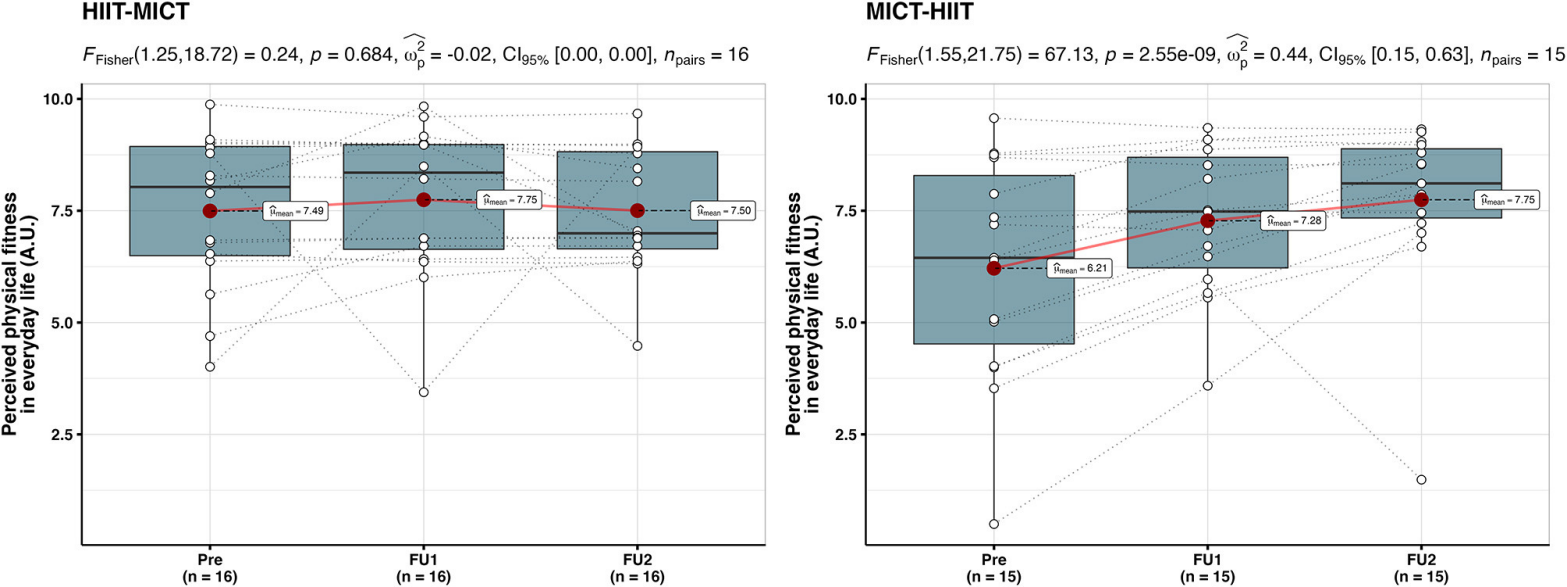
Within-group between baseline (Pre), follow-up I (FU1), and follow-up II (FU2) in the HIIT-MICT and MICT-HIIT groups for perceived physical fitness in everyday life. Boxplots along with the individual data points are shown. The dotted lines connect the same participant, displaying the individual changes. The red dots display the average of each time point.

For perceived physical fitness in everyday life (Figure 6), no significant effects were observed in the HIIT-MICT group {*F*_(1.25, 18.72)_ = 0.24, *p* = 0.68, ω^2^ = −0.02, 95% CI = [0.00; 0.00]}. The MICT-HIIT group, however, showed a significant main effect {*F*_(1.31, 18.41)_ = 6.74, *p* = 0.013, ω^2^ = 0.08, 95% CI = [0.00; 0.28]}. Pairwise *post-hoc* comparisons revealed a significant difference after MICT (+ 59.7 ± 158%, *p* = 0.003, from baseline to follow-up I), but not after HIIT (+ 9.7 ± 33.7%, *p* > 0.05, from follow-up I to follow-up II).

**Figure 7:**
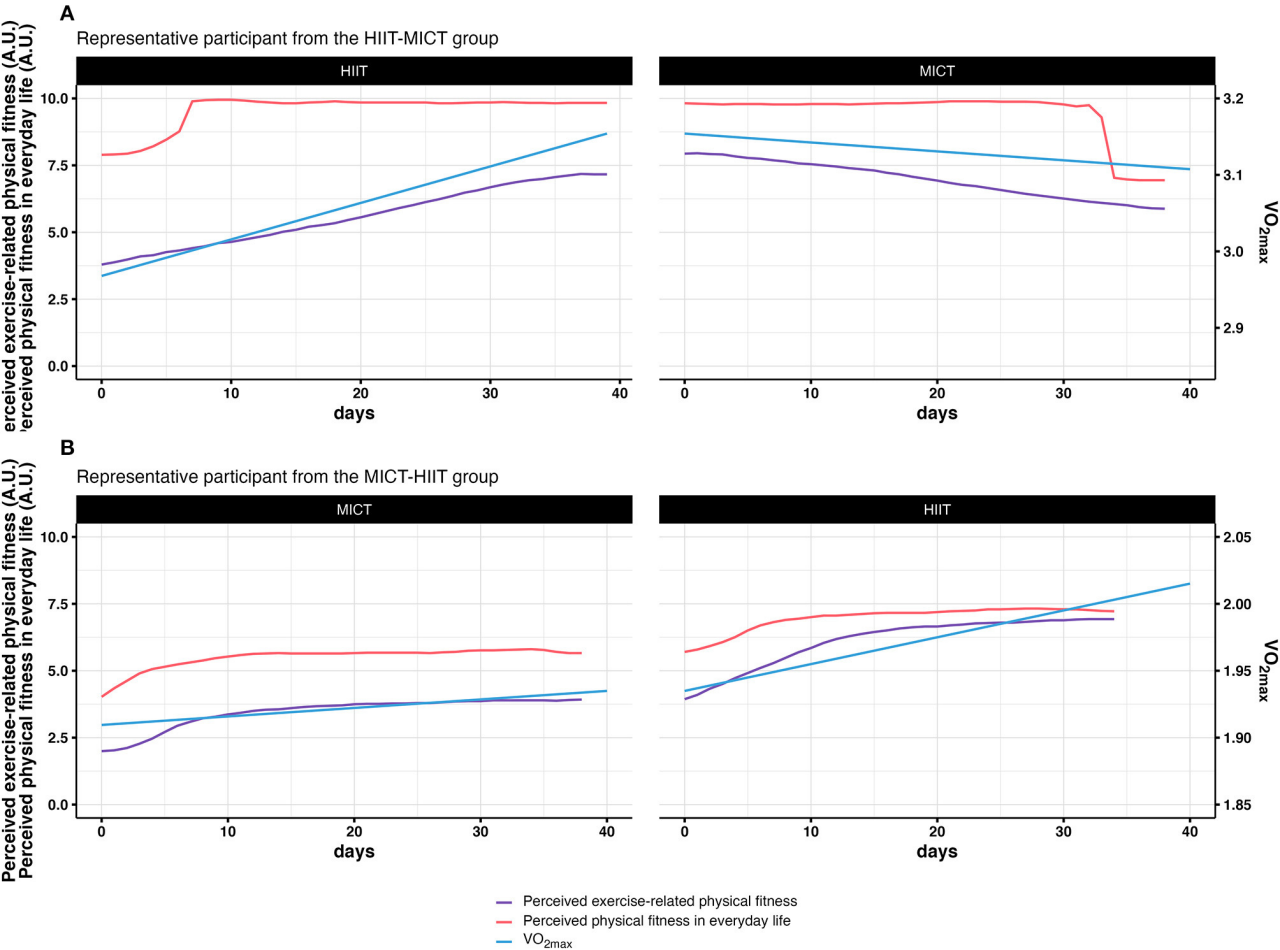
Integration of physiological and subjective measures of fitness. **(A)** displays the integrated results for a representative particpant in the HIIT-MICT group. **(B)** displays the integrated results for a representative particpant in the MICT-HIIT group.

Figure 7 displays an integration of physiological and subjectively perceived fitness measures in two participants who are archetypical representatives of averaged developments.

**Figure 8:**
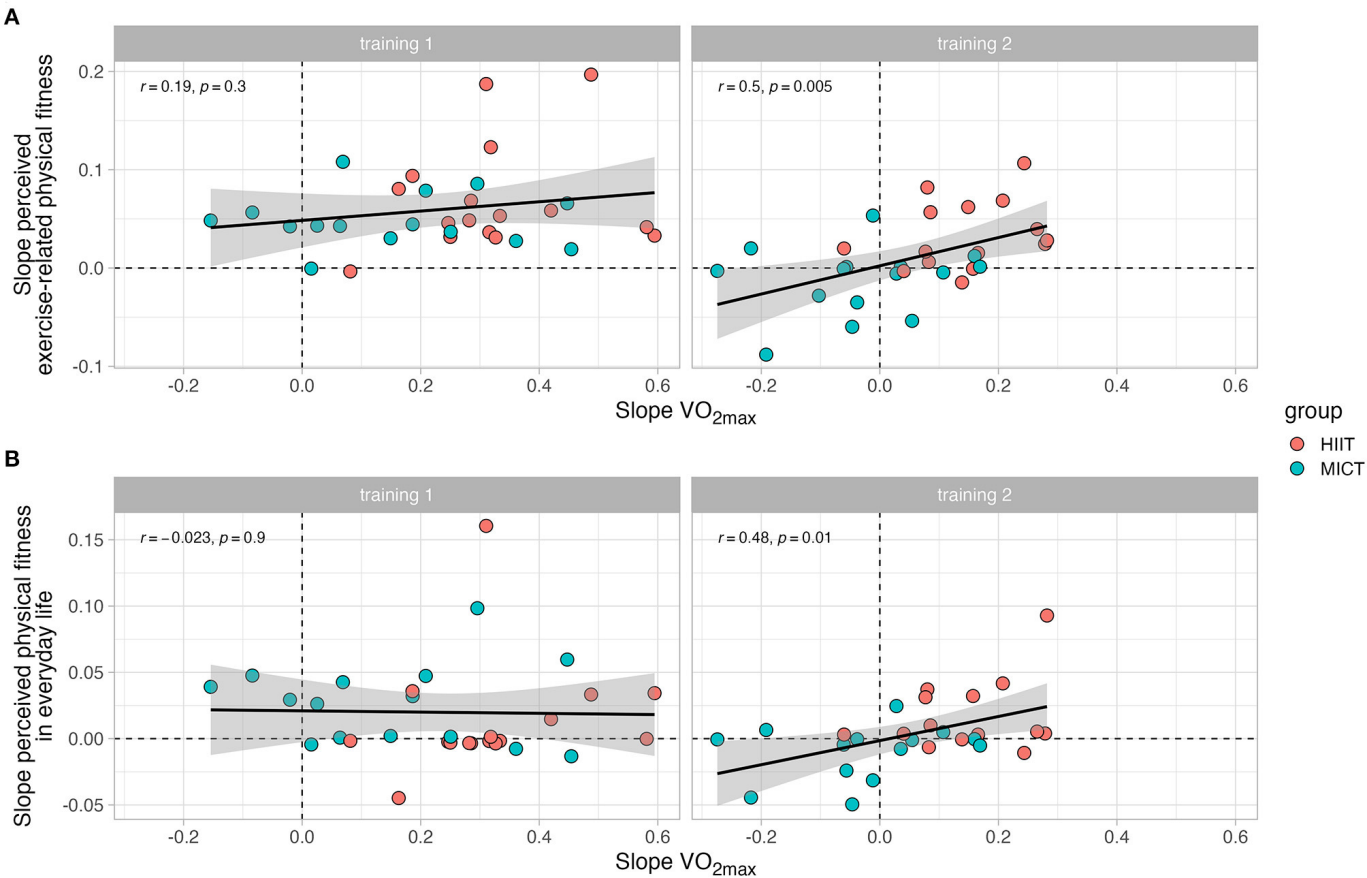
Comparison of the rate of change (i.e., slopes) in VO_2max_ with the rate of change in subjective scales of fitness. **(A)** displays the association between the slopes associated with VO_2max_ and the slopes associated with perceived exercise-related physical fitness. **(B)** displays the association between the slopes associated with VO_2max_ and the slopes associated with perceived physical fitness in everyday life. Each panel is separated into training 1 (from baseline to follow-up I) and training 2 (from follow-up I to follow-up II).

**Figure 9:**
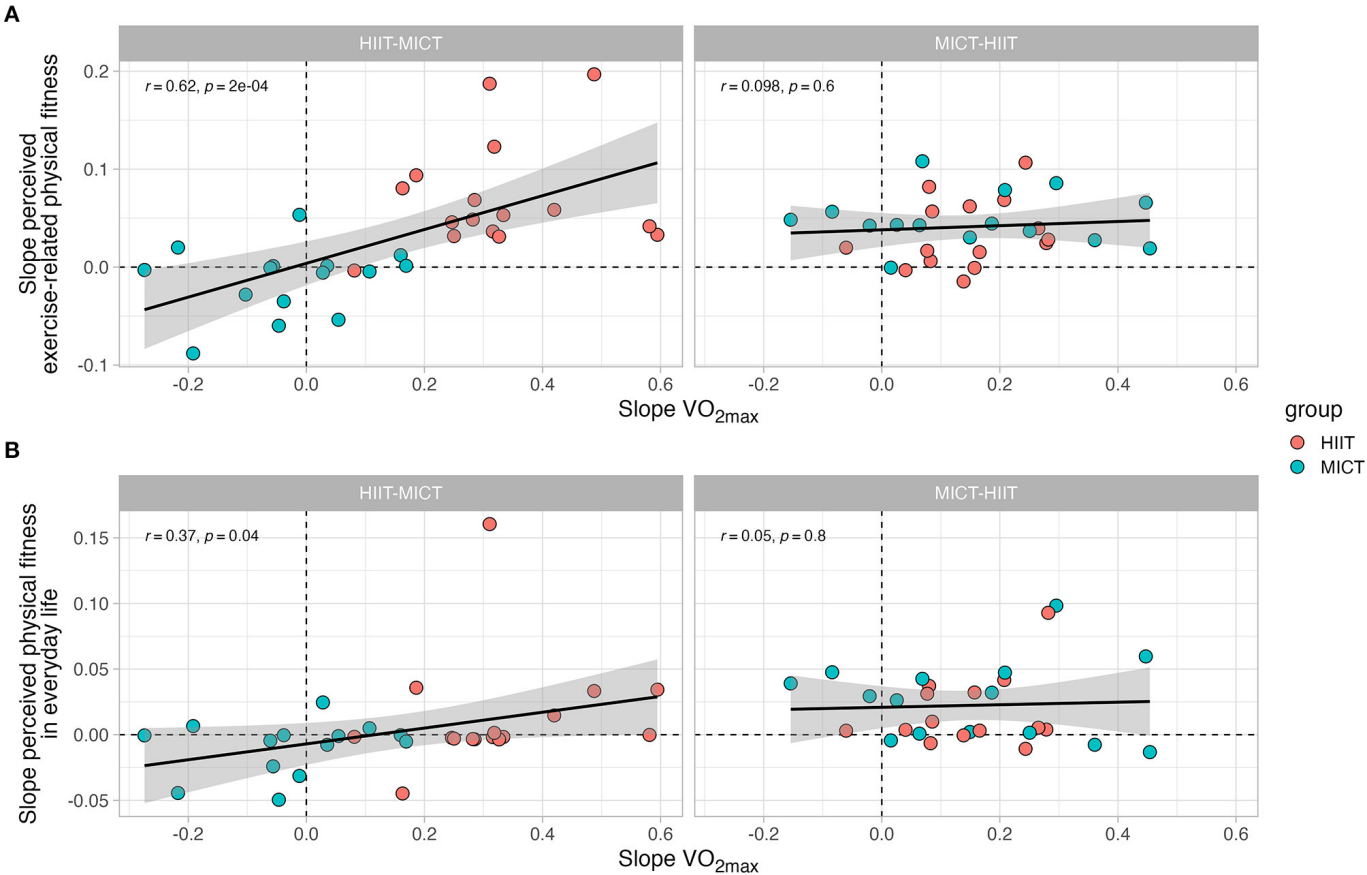
Comparison of the rate of change (i.e., slopes) in VO_2max_ with the rate of change in subjective scales of fitness. **(A)** displays the association between the slopes associated with VO_2max_ and the slopes associated with perceived exercise-related physical fitness. **(B)** displays the association between the slopes associated with VO_2max_ and the slopes associated with perceived physical fitness in everyday life. Each panel is separated into each training group (i.e., HIIT-MICT and MICT-HIIT).

A slope comparison between the values from baseline to follow-up I (first training period) and from follow-up I to follow-up II (second training period) was performed in order to analyze the association between VO_2max_ changes and changes in subjective fitness perceptions after training (Figure 8). A significant correlation was observed in the second training period for both the relationship between VO_2max_ and the perceived exercise-related physical fitness (*r* = 0.5, *p* = 0.005), and the relationship between VO_2max_ and the perceived physical fitness in everyday life (*r* = 0.48, *p* = 0.01). No significant correlations were observed during the first training period, indicating a different rate of change between the variables. However, the slope diagrams show a consistent pattern after the second training period, meaning that the correlation between VO_2max_ and subjective fitness (in both dimensions) was in most cases clearly higher in HIIT compared to MICT. Additionally, a slope comparison between the sequence of the training modes was performed (i.e., HIIT-MICT and MICT-HIIT) (Figure 9). A significant correlation was observed in the HIIT-MICT group for both the perceived exercise-related physical fitness (*r* = 0.62, *p* < 0.001) and perceived physical fitness in everyday life (*r* = 0.37, *p* = 0.04) with VO_2max_. No significant correlations were observed in the MICT-HIIT group.

## Discussion

Dose-response effects of exercise on health and physical fitness are well-established on a physiological level (Fiuza-Luces et al., 2013, 2018; Milanović et al., 2015; Gomes-Neto et al., 2017; Maturana et al., 2020). What is less known is whether physiological adaptations to exercise that occur across the period of an intervention are subjectively experienced in a comparable way. PA (and hence exercise) has been deemed a “complex and multidimensional behavior” (Pettee Gabriel et al., 2012) that incorporates a multitude of correlates and determinants (Rhodes and Nigg, 2011; Bauman et al., 2012). In order to analyze the response to exercise appropriately, a more holistic, interdisciplinary, and biopsychosocial perspective is needed (John et al., 2020). In this sense, the aim of our study was to assess how individuals *physiologically respond* to exercise and how those responses are *subjectively experienced and perceived* over time. The underlying assumption of our study was that for exercise interventions to be successful and sustainable, physiological benefits, such as the improvement of physical fitness, must also be subjectively experienced as beneficial. By employing a mixed-methods approach, we set out to describe trends in the development of physiological and subjectively experienced fitness and to see how these two dimensions relate to each other.

Contributing to the ongoing discussion on the viability of HIIT for public health strategies from a different perspective (Biddle and Batterham, 2015), we compared the physiological and subjective fitness-related responses to two distinct endurance-based training programs (i.e., HIIT and MICT). In this regard, we also wanted to know whether the sequence of different training modes (i.e., HIIT-MICT and MICT-HIIT) has an impact on physiological and subjective fitness-related responses to training.

Our analyses have shown that VO_2max_ and perceived exercise-related physical fitness significantly improved both in the HIIT-MICT and the MICT-HIIT group indicating that inactive young adults can physiologically and subjectively benefit from a structured exercise intervention. In general, however, HIIT yielded greater effects for VO_2max_ than MICT, which is in line with recent research on healthy young to middle-aged adults (Milanović et al., 2015). The slope comparisons show that after the completion of the second training period, the correlation between subjective and physiological fitness is generally higher with regard to the HIIT mode. However, the sequence of training modes nevertheless had an impact on the physiological and subjective fitness outcomes. While the MICT-HIIT sequence was associated with continuous improvement in physiological fitness, the HIIT-MICT sequence was associated with increases only during the first training period. These results suggest that the sequence of training modes can have a substantial impact on the physiological response to exercise. MICT is a key training stimulus for long-term training (Bishop et al., 2019) and an important training mode in metabolic health (Maturana et al., 2020). However, the higher efficacy promoted by HIIT highlights the importance of controlling the exercise intensity for CRF improvements, at least in the short-term training (MacInnis et al., 2019). Similar tendencies were observed for perceived exercise-related fitness with a significant main effect for the HIIT-MICT group, as well as for the MICT-HIIT group. Interestingly, the MICT-HIIT group showed a significant increase in perceived exercise-related fitness across both training periods, while for the HIIT-MICT group we observed a significant increase only in the first training period. For the second training period in this group, we even found a statistically significant decrease in perceived exercise-related physical fitness. In our study, participants were not only physiologically untrained, but also the self-perceived levels of fitness tended to be in the lower half of the scale at the beginning of the exercise intervention. It is therefore not surprising that starting to exercise therefore yielded improvements in both physiological and perceived exercise-related physical fitness independent of exercise modality since for inactive or sedentary samples, even small increases in volume or intensity of PA can increase physical fitness (Maturana et al., 2021). The effects of HIIT were perceived as stronger, independent whether the group started with HIIT or MICT. For the group that started with MICT in the first training period, the subjectively perceived fitness-enhancing effects of the first training period increased further when they completed the physiologically more effective HIIT in the second training period. On the other hand, MICT is experienced as less effective regarding individual fitness enhancement, particularly if this training mode is performed after a physiologically and subjectively highly effective period of HIIT. Training moderately after having trained in orientation to a HIIT regime obviously has the effect that individual fitness is experienced even more negatively than the physiological situation actually has developed. Despite the reported developmental trends in VO_2max_ and perceived exercise-related physical fitness, regular exercise does not necessarily lead to a substantial increase in the subjective perception of physical fitness in everyday life. This may be because our sample consisted of inactive, yet young and healthy adults, who had no substantial physical impairments and thus were also able to participate in our exercise intervention. Accordingly, most participants rated their subjective fitness levels in everyday life in the upper half of the scale and generally showed little intra-individual variation over time.

In general, our results clearly show high degrees of variability within- and between-individuals in the subjective fitness parameters. Even though the metabolic stress imposed by each exercise training program was adjusted to be the same for all individuals (i.e., MICT was prescribed within the moderate-intensity domain and HIIT was prescribed within the severe-intensity domain), the perceived development of fitness showed to be heterogeneous.

When interpreting the results of our study, several strengths but also limitations should be considered. First, while there are intervention studies that use mixed-method approaches (with the qualitative assessments having a focus rather on attitudes toward PA and exercise than on physical fitness) this is, to our knowledge, the first paper that focuses explicitly on the development of physical fitness as a response to exercise over time from both a physiological and a subjective-experiential perspective. By integrating “objectively” measurable fitness data and quantified constructions of retrospective and subjective experiences, our results may help better understand the relationship between exercise mode and individual responses to exercise. Second, by comparing HIIT to MICT and the respective sequences of the training modes, we aimed to contribute to the discussion on the viability of HIIT for public health measures. By focusing on the physiological and subjectively perceived development of physical fitness depending on different training modes, we hope to add another dimension to the discussion.

While we believe that our mixed-method approach might offer a more integrated perspective on and a broader understanding of the development of physical fitness in a structured exercise setting, our study is also associated with some shortcomings and limitations. First, with 31 participants and two experimental arms (HIIT-MICT and MICT-HIIT) our sample size is not particularly large. However, considering the explorative approach of the iReAct study and the fact that it was designed as a 15-week intervention (including 3 weeks of diagnostics and 12 weeks of training), time, economic, and personnel constraints limited the possible inclusion of further participants. Moreover, the last assessment wave (including nine more participants) had to be terminated early due restrictions during the early phase of the COVID-19 pandemic. Second, our study did not include an inactive control group that did not exercise at all. However, this was an intentional choice as particularly the effects of a sequential order of MICT and HIIT were of interest and the HIIT-MICT group and the MICT-HIIT group were thus used as controls for each other. Third, our study was designed to include young inactive adults from all social milieus. However, maybe due to the extraordinary time expenditure required for the participation, mainly students who were enrolled at the university signed up for the study. Although some participants were already employed, those were only few. In general, our findings relate in particular to a well-educated sample of young inactive adults. Fourth, limitations with regard to our methodological approach have to be considered. Our goal was to integrate physiological and subjectively experienced physical fitness measures. One of the main limitations in this regard is the potential lack in correspondence between our physical fitness proxies. The physiological variable for physical fitness in our study is VO_2max_, which refers primarily to CRF. On the other hand, our approach to collect experiential data on physical fitness was based on an open understanding of physical fitness, with the only specification being that we distinguished between exercise-related physical fitness and physical fitness in everyday life. Other than that, participants were free to choose their own definition of the concept. The high degree of specificity for the physiological outcome on the one hand, and the openness with regard to the subjectively experienced outcomes on the other, may have led participants to refer to different dimensions of fitness (e.g., including musculoskeletal fitness or flexibility). In addition, with regard to the assessment of subjective experiences, we relied on retrospectively constructed (i.e., reconstructed) accounts. While those reconstructed experiences do not display perceptions during or immediately after an exercise cue, they are evaluative judgments that can express participants' current (i.e., after the exercise intervention at the time of data collection) attitudes, for example toward different training modes. These attitudes, in turn, are based on previous experiences that are comparatively evaluated (e.g., with regard to different training modes) in hindsight. We are aware that the sequential analysis of training modes as presented here is rather uncommon, however, we think that our results indicate that this particular comparison might have practical implications. For example, our results show that training moderately after having trained with HIIT for a while, might be less beneficial both physiologically and psychologically. Yet, we have to acknowledge that our study provided a highly structured intervention setting, in which participants had to exercise on a regular basis, had to adhere to a minimum amount of training sessions, were monitored in their progress, and participated in a wide range of biopsychosocial assessments. This “clinical” procedure does not necessarily translate to PA, exercise, and sport in everyday life. Subjective experiences of feeling fit probably differ across contexts, activities, and, as we have indicated, training type. Additionally, while we assume that feeling fit might be one motivational determinant of regular exercise and PA, we are aware that PA is a complex phenomenon that is associated with a multitude of determinants and correlates and lab-based exercise interventions may be able to only account for some them. The question therefore remains, how sustainable our exercise intervention was. Unfortunately, we cannot make any statements as to which extent our study actually motivated participants to remain active after they completed the 15 weeks, since the major goal was to assess more or less immediate individual biopsychosocial responses to exercise as well as subjectively reconstructed experiences of those responses.

We are convinced that our findings provide a new and important perspective on how physical fitness develops according to the prescribed training modes and how changes are subjectively experienced. Future research might build on the strengths of our study and aim to counteract the potential limitations we have described. In particular, when it comes to relating physiological fitness measures to subjective experiences of physical fitness, future studies might aim to go into more depth and include more measures of physiological fitness (e.g., musculoskeletal fitness or flexibility) and more differentiated measures of perceived physical fitness (e.g., by asking specifically about perceived cardiorespiratory fitness or perceived strength). Moreover, future studies might aim to provide deeper insights and explanations for the observed trends by assessing individual and subjective accounts of physical fitness from a mainly qualitative perspective, as inactive young adults “respond differently to different forms of exercise and the factors that influence participation in interval or continuous exercise are far more complex than can be captured by quantitative methodologies alone” (Stork et al., 2020, p. 10). Additionally, future studies might try and follow-up on participants for an extended period of time, in order to see how effective the exercise intervention actually was in terms of adoption, adherence, maintenance, and retention rates (Willinger et al., 2021).

## Conclusion

It is well-established that regular exercise fosters substantial health-enhancing benefits. Yet, physical inactivity and sedentariness are predominant and uptrending phenomena of our time. We assume that PA and exercise are more likely to be pursued in the long run if they promote not only objective health and fitness benefits, but if those benefits can also be subjectively experienced. Exemplifying this in the context of physical fitness, our results indicate that young inactive adults can benefit from regular exercise with regard to physical fitness outcomes both physiologically and subjectively. However, the choice of exercise modes and their sequence can make a substantial difference in measured and perceived physical fitness. This should be taken into consideration by exercise researchers and PA and public health practitioners alike. In addition, our results have shown that the ways in which young inactive adults respond physiologically and experientially to exercise vary inter-individually emphasizing the necessity of individualized approaches to exercise prescriptions. While for most young inactive adults HIIT seems to foster physiological and perceivable fitness benefits, one-size-fits-all approaches cannot be the solution to such a complex phenomenon as exercise.

## Data Availability Statement

The raw data supporting the conclusions of this article will be made available by the authors, without undue reservation.

## Ethics Statement

The studies involving human participants were reviewed and approved by Ethics Committee of the Medical Faculty University Tübingen. The patients/participants provided their written informed consent to participate in this study.

## Author Contributions

AT and AN contributed to the conception and overall design of the iReAct study and the funding acquisition. AT, AN, HG, and FMM contributed to the conception and design of the substudy for this paper. HG conducted the biographical mappings and drafted the first version of the manuscript. FMM collected physiological data, performed the statistical analyses, and wrote sections of the manuscript. All authors contributed to critical manuscript revision and read, edited, and approved the submitted version.

## Conflict of Interest

The authors declare that the research was conducted in the absence of any commercial or financial relationships that could be construed as a potential conflict of interest.

## Publisher's Note

All claims expressed in this article are solely those of the authors and do not necessarily represent those of their affiliated organizations, or those of the publisher, the editors and the reviewers. Any product that may be evaluated in this article, or claim that may be made by its manufacturer, is not guaranteed or endorsed by the publisher.
