# Supplementary figures and images for: Does Becoming Fit Mean Feeling (f)it? A Comparison of Physiological and Experiential Fitness Data From the iReAct Study
Source: Front Sports Act Living. 2021 Sep 1;3:729090. doi: 10.3389/fspor.2021.729090 (PMC8440924; doi:10.3389/fspor.2021.729090)

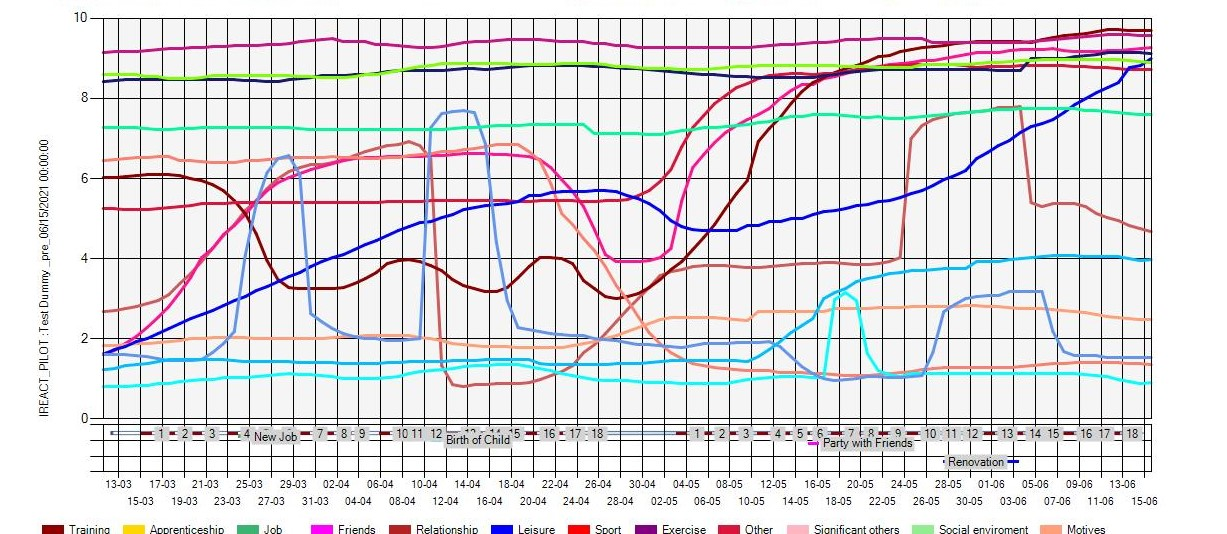

Supplement: Supplementary file 1 [file Image_1.TIF]
